# Marine litter is a notable challenge to crabs in highly impacted mangrove areas: a case study in Guanabara Bay, SE Brazil

**DOI:** 10.1007/s10661-026-15341-x

**Published:** 2026-04-29

**Authors:** Eduardo Vianna de Almeida, Priscilla de Oliveira Gomes dos Santos, Luciana Pereira Torres Chequer, Viviane Duarte Gonçalves, Tainá Stauffer, Jorge Elias Rage-Aboud, Carla Muniz Sabino, Gabriela Verônica Buraschi, Susana Beatriz Vinzon

**Affiliations:** 1 Laboratório de Carcinologia, Departamento de Zoologia, Instituto de Biologia, Universidade Federal do Rio de Janeiro (UFRJ), Rio de Janeiro, Brazil; 2 Programa de Pós-Graduação em Dinâmica dos Oceanos e da Terra, Universidade Federal Fluminense (UFF), Niterói, Brazil; 3 Programa de Pós-Graduação em Biodiversidade e Biologia Evolutiva, Universidade Federal do Rio de Janeiro (UFRJ), Rio de Janeiro, Brazil; 4 Unidade Multiusuário de Análises Ambientais (UMAA), Universidade Federal do Rio de Janeiro (UFRJ), Rio de Janeiro, Brazil; 5 Programa de Pós-Graduação em Zoologia, Museu Nacional – Universidade Federal do Rio de Janeiro (UFRJ), Rio de Janeiro, Brazil; 6 Laboratório de Dinâmica de Sedimentos Coesivos (LDSC), Instituto Alberto Luiz Coimbra de Pós-Graduação e Pesquisa de Engenharia, Universidade Federal do Rio de Janeiro (COPPE - UFRJ), Rio de Janeiro, Brazil

**Keywords:** Marine pollution, Solid waste, Plastic debris, *Ucides cordatus*, Fiddler crabs

## Abstract

Mangroves represent a particularly vulnerable coastal ecosystem to the problem of marine litter. However, research addressing the impact of macroscopic litter on mangrove crabs remains limited. The aim of this study was to characterize diversity, abundance, and size of the semi-terrestrial crabs in a mangrove swamp heavily affected by macroscopic marine litter, in Southeastern Brazil. It was also aimed at verifying any potential correlations between crab parameters and the percentage of substrate covered by marine litter. Quadrats of 3.0 m × 3.0 m were used in three subzones of the intertidal zone (lower, middle, and transition—between upper intertidal and supralittoral). The litter was characterized after collection, and marine litter coverage was assessed using 1.0 m × 1.0 m photographs, which were analyzed using CPCe software. The fiddler crabs were analyzed by catch and release and the crab *Ucides cordatus* by examination and counting of burrows. The abundance of marine litter ranged from 69.6 to 120.3 items.m^−2^, and the marine litter coverage varied between 26.0 and 100.0%. The diversity and abundance of crab species was found to be low (0.32 to 1.46 bits; 0.1 to 2.0 ind.m^−2^). The marine litter coverage had a significant negative effect on crab abundance (*R* = −0.63; *p* = 0.03). In addition, a strong and significant influence of marine litter coverage on the size of both *U. cordatus* (Pearson, *R* = 0.83; *p* = 0.02) and *Minuca* spp. (only females; Pearson, *R* = 0.80; *p* < 0.001) was observed.

## Highlights

- Marine litter covered 26 to 100% of the mangrove substrate.
- Marine litter was often found stuck to the roots, crab burrows and substrate.
- Areas with more litter had significantly less crabs.
- Areas with more litter had larger *Ucides cordatus*.

## Introduction

Mangroves are transitional coastal ecosystems between land and sea, located in tropical and subtropical regions. They are tidal ecosystems with predominantly fine, hypoxic sediments, a high concentration of organic matter, and high biological productivity, supporting plant and animal species adapted to the typical diurnal variations in salinity. Mangroves support species of economic interest such as fish, mollusks, and crustaceans, and are an ecosystem used by many species as breeding and spawning grounds (Checon et al., 2023). Despite their importance, mangroves are among the ecosystems most threatened and affected by anthropogenic activities, being the destination of distinct types of pollutants like marine litter, especially garbage (Martin et al., 2019).

The low cost of plastic production, its high durability and typically low density, as well as its widespread use in human activities, are some of the factors related to its high frequency among marine litter (Andrady & Neal, 2009), including in coastal ecosystems such as mangroves (Martin et al., 2019). Several species of mangrove trees have aerial roots (e.g. rhizophores and pneumatophores) that reduce water flow and facilitate the retention of sediments. These same roots may also retain marine litter (Ivar do Sul et al., 2014; Martin et al., 2019; Riascos et al., 2019). As a result of failures in distinct parts of society, plastics have entered aquatic environments in large quantities, either through continental runoff or wind, and are easily transported beyond their point of origin (Van Sebille et al., 2020). As much of the plastic debris is not biodegradable, it accumulates in mangroves and other coastal systems (Kubota et al., 2005; Luo et al., 2021) and can cause various negative effects on organisms, including crabs (Pisani et al., 2022). This global pattern is particularly evident along the Brazilian coast, where macro- and microscopic marine litter pollution represents a severe environmental problem. It is estimated that Brazilian municipalities contribute 3.44 million metric tons of poorly managed plastics to the environment each year, of which 1.62 million metric tons are in the coastal environment (Alencar et al., 2023). Notably, Guanabara Bay, the present study area, together with the Prata River, has been identified as one of the primary sources of marine litter entering the Brazilian coast (Alencar et al., 2023).

Despite the rising cognizance of plastic contamination in mangroves, extant research has predominantly centered on distribution and abundance of marine litter, thereby leaving the exploration of biological interactions and species-level effects, particularly on crabs, largely unexplored (Costa et al., 2025). As reviewed by Pisani et al. (2022), studies examining the impacts of marine litter on crabs have predominantly focused on microplastics, reporting effects such as reductions in feeding rate, body mass, and metabolic activity. Conversely, research addressing macroscopic litter remains limited, accounting for only approximately 2.5% of the available literature, with mangroves ranking relatively low among the ecosystems examined. The paucity of research on the subject is evidenced by the inconsistent ecological outcomes reported in the existing studies.

Previous studies have identified a negative correlation between the density of fiddler crab burrows and the marine litter coverage (Bulow & Ferdinand, 2013). However, more recent research has not found a significant relationship between the distribution of macroscopic litter and crab population parameters (Riascos & Gomez, 2023). Other studies have documented negative ecological effects associated with macroscopic litter, including the physical blockage of crab burrows (Iribarne et al., 2000; Luo et al., 2021). Taken together, these findings indicate that empirical evidence on interactions between macroscopic marine litter and mangrove crabs remains sparse and fragmented. Consequently, further research is necessary to elucidate the impact of macroscopic litter on crab populations and the associated ecological processes within mangrove ecosystems. Therefore, the objective of this study was to characterize the populations of semi-terrestrial crabs in a mangrove swamp in southeastern Brazil (Guanabara Bay, South Atlantic Ocean), which is highly affected by anthropogenic macroscopic litter. It also aimed to carry out a qualitative and quantitative analysis of litter to verify possible relationships between crab population parameters and the percentage of substrate covered.

## Materials and methods

### Study area

**Fig. 1:**
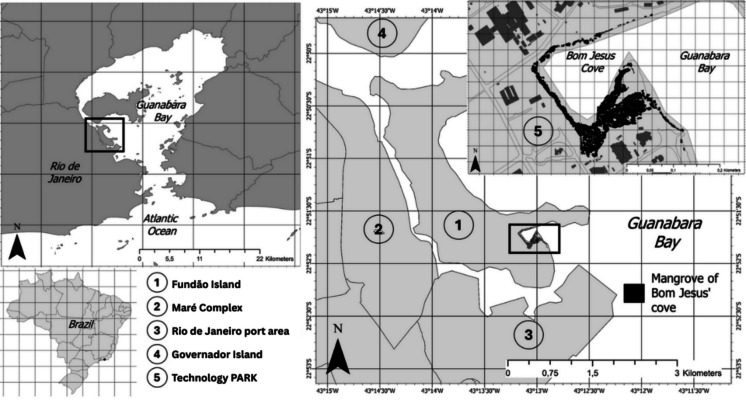
Location of the Bom Jesus Cove mangrove swamp on the Fundão Island, Rio de Janeiro, Brazil

In Brazil, mangroves occur from Cape Orange, in Amapá (04° N, 50° W), to the city of Laguna, in Santa Catarina (28° S, 48° W), and are subject to high latitudinal variations in environmental conditions (Schaeffer-Novelli et al., 1990; SMAC, 2000). The present study was conducted in the mangrove swamp of Bom Jesus Cove (MBJC) (Fig. 1), in the western portion of Guanabara Bay (SE, Brazil), a region with the most adverse environmental conditions within the bay (Borges et al., 2014). The western part of the bay has a high urban and industrial concentration, with substantial amounts of anthropogenic litter and untreated wastewater, especially domestic sewage (Soares-Gomes et al., 2016). The strategic positioning of MBJC on Fundão Island, near the research laboratories associated with the *Orla sem Lixo* Project, renders the mangrove forest a suitable locale for investigative approaches, thus establishing it as a model area. Near the MBJC are the Port of Rio de Janeiro and the Maré complex, one of the largest slums in Latin America, with approximately 140,000 inhabitants (Redes da Maré, 2019). In MBJC, access is restricted and fishery in *U. cordatus* is rare.

The mangrove is near to the mouth of the Cunha Channel, characterized by low-energy hydrodynamics, which contributes to the accumulation of fine sediments and the formation of extensive areas with low dissolved oxygen (Fistarol et al., 2015). The region is under the influence of semi-diurnal micro-tides, with a maximum amplitude of ± 1.4 m. However, due to stochastic climatic events in the Atlantic Ocean, meteorological tides can occur that increase or decrease the amplitude of sea level variation. This phenomenon, frequently induced by variations in pressure and alterations in wind patterns (Borba et al., 2023), has the potential to induce flooding in certain regions of the supralittoral zone of the Fundão Island study area (Dias & Oliveira, 2024) and may impact the dynamics of floating debris.

In Guanabara Bay, the climate is tropical humid, with more rainfall in summer (Dec–Mar) and less in winter (Jun–Sep) (Felippo & Figueiredo-Jr, 2012) . The mean air temperature at the weather station nearest to MBJC (São Cristóvão neighborhood) in 2022 was 30.0 ± 4.1 °C (Alerta Rio, 2025). The maximum temperature was recorded as 39.1 °C in March, while the minimum was 19.8 °C in September. In the MBJC, the predominant vegetation is black mangrove (*Avicennia schaueriana* Stapf & Leechm. ex Moldenke). However, it is possible to find some specimens of red mangrove (*Rhizophora mangle* L.), beach cotton (*Talipariti pernambucense* (Arruda) Bovini), and the invasive fern *Acrostichum danaeifolium* Langsd. & Fisch.

### Sampling strategy

A transect measuring 70 m in length was extended from the supralittoral to the lower intertidal zone. Sampling quadrats measuring 3.0 m × 3.0 m (9.0 m^2^ each) were utilized at consistent points across three subzones: (1) Lower Zone (LZ)—located near the waterline, subject to daily tidal flooding, and dominated by dense stands of black mangrove pneumatophores; (2) Middle Zone (MZ)—positioned higher in the intertidal gradient, inundated mainly during neap and spring tides, with fewer pneumatophores; and (3) Transition Zone (TZ)—forming the upper boundary between the intertidal and supralittoral zones, seldom flooded except during meteorological tides. For the analysis of crabs and marine litter, two quadrats per subzone were employed in each campaign. It was observed that all the quadrats exhibited partial (> 50.0% cover) or complete shading by black mangrove trees.

### Sediment analysis

In each subzone, three samples of surface sediment (up to 10 cm) were collected in May 2022 for analysis of abiotic variables and organic matter content. The parameters examined included water content (%), organic matter content (%), carbonate (mg.kg^−1^), and granulometric analysis using a laser granulometer (Malvern, Hydro 2000 UM).

### Macroscopic marine litter assessment

The assessment of macroscopic marine litter was conducted through two approaches: the estimation of marine litter covering the substrate via photographic documentation, and a qualitative-quantitative analysis following collection of marine litter. The estimation by photographs was assessed in May and August 2022 in the sample quadrats, with the aid of a 1.0 m × 1.0 m frame. Nine photographs were taken per quadrat using an Olympus TG-6 camera. The software Coral Point Count with Excel extensions (CPCe; Kohler & Gill, 2006) was originally developed for the purpose of determining coral coverage using photographs of transects. In the present study, the codes of this software were adapted to analyze the macroscopic marine litter coverage. The results were expressed in percentage and area (m^2^).

The estimation of marine litter abundance by collection (in items per m^2^) was made near to the crab sample area, also in 3.0 m × 3.0 m quadrats, in each subzone (LZ, MZ, and TZ). The collection took place in July 2022 and was carried out with the support of the local fishing community. The collected litter was transported to the laboratory where the items were counted and identified by type.

### Semi-terrestrial crab study

#### Preliminary surveys

The initial expeditions were conducted in September and November 2021 and March 2022. The objective was to enhance familiarity with the study area and undertake a census of crab species, mainly with photographic documentation. An active investigation was conducted of the substrate and burrows, on habitats between roots, on shrub and tree strata, and under marine litter. Some specimens were captured, rendered insensitive through freezing, and subsequently transported to the laboratory for confirmation of species. The following bibliography was used for the identifications: Abele (1992), Melo (1996), Niem (1996), Bezerra (2012), Masunari et al. (2020), and Stauffer (2024). The research was conducted in accordance with the provisions of license number 78819-1 (SISBio—ICMBio/Brazilian Ministry of the Environment—MMA).

#### Crab population monitoring

Two field surveys were conducted in May and August 2022. Small species, such as fiddler crabs, were analyzed by capture and release. The larger crab, *Ucides cordatus* (Linnaeus, 1763) (“uçá crab”), was assessed by analyzing and counting burrows. In the analysis of fiddler crabs, the specimens were captured, identified, and measured at the collection site (carapace width—CW) using digital calipers with a precision of 0.01 mm. Manual collection was conducted by two collectors for a standardized effort of 15 min per quadrat per field survey. The collectors used metal spatulas, focusing on the initial 10.0 cm of substrate depth. Fiddler crabs were captured both on the substrate and within the entrances and initial sections of burrows. The utilization of spatulas for the obstruction of burrow entrances was a common practice, with the objective of impeding the progression of the animals into more profound burrow sections.

The uçá crab population was analyzed as proposed by Pinheiro and Almeida (2015). In each quadrat, burrows were counted and classified (active, closed, or inactive), and the width of the burrow entrance was measured using an adapted caliper (Schmidt et al., 2008). It is important to note that meticulous attention was devoted to the process of avoiding the duplication of counts, thereby ensuring the integrity of the data collection. The care was related to the direction of counting (unidirectional) and the assistance of two observers, who remained next to the quadrat. About burrow classification, active burrows were defined as those showing signs of recent activity, such as the presence of fresh tracks, sediment, or feces near the entrance. Closed burrows were defined as those with a clear raised 'mud cover' made by the crab itself for ecdysis. Burrows that contained no tracks, fresh mud, or feces and were blocked by plant debris or spider webs were considered inactive (unoccupied). To estimate the carapace width of uçá crabs, after measuring the burrows, we used the equation formulated by Pinheiro (2006). This equation is expressed as follows: CW = 13.21 + 0.9602*BD, with CW representing carapace width and BD denoting burrow diameter, with results expressed in mm.

The crab abundance, or population densities, was obtained by dividing the number of crabs by the area of each quadrat (9.0 m^2^), expressed in individuals per m^2^ (ind.m^−2^). For *U. cordatus*, only active and closed burrows were included in this calculation. It is worthy of note that certain authors have reported the existence of *U. cordatus* burrows belonging to one or two exits (see, for example, Pinheiro & Almeida, 2015). In the context of studies employing burrow counts, the phenomenon in question has the potential to yield misleading results. Consequently, prior to and during field studies, an investigation was conducted into the burrow exits at the MBJC. In our study area, no burrows with two exits were found. Therefore, we consider that each active or closed burrow entrance represented a single gallery inhabited by a crab.

### Data analysis

Tables and histograms were produced using the Excel for Windows software. Boxplots and statistical analyses were conducted using the Jamovi software (The Jamovi Project, 2022). The diversity calculation, expressed in bits (Shannon Index), was conducted in the Primer 5 software, employing the recorded richness and abundance of crabs in each quadrat. The densities of the dominant species and those that occur sporadically in the quadrats, were considered. The male-to-female ratio of fiddler crabs was evaluated using the chi-square (χ^2^) test to ascertain whether a significant difference existed. This parameter was only able to be calculated for *L. uruguayensis*. This was not the case for *Minuca* spp., which comprise a group of different species.

A comparative analysis was conducted of the sediment properties, population densities, and crab sizes across the three subzones and the two field surveys. The normality and homogeneity of the data were verified by the Shapiro–Wilk and Levene tests, respectively. For the purpose of analysis of the subzones, the one-way analysis of variance (ANOVA) test was employed, followed by Tukey’s test (parametric statistics) or the Kruskal-Wallis test, followed by multiple comparisons of the DSCF test (non-parametric statistics). To make a comparison between the field surveys, the t-test (parametric) or the Mann–Whitney test (non-parametric) were employed.

The present study investigates the correlations between crab diversity (bits), population densities (ind.m^−2^), specimen size (including estimated CW of *U. cordatus*) and the percentage of substrate covered by marine litter. Considering the outcomes of the preliminary tests (Shapiro–Wilk and Levene tests), it was determined that Pearson’s correlation coefficient (parametric analyses) would be employed. The classification of the correlations was then based on the approach delineated by Vieira (2020). All statistical tests were conducted with a 95% confidence interval (*p* ≤ 0.05).

## Results

### Sediment analysis

The sediments of all three subzones were poorly sorted, with the majority classified as sandy-muddy gravel, according to Folk (1954). The intertidal lower and middle zones exhibited significantly higher water content (LZ 50.5 ± 13.3%; MZ 39.9 ± 9.7%) and higher percentage of organic matter (LZ 18.6 ± 7.7%; MZ 11.6 ± 2.6%) (ANOVA, *p* < 0.05). The water content of the transition zone was 15.2 ± 2.2%, and the organic matter content was 3.5 ± 0.4%.

### Marine litter analysis

**Fig. 2:**
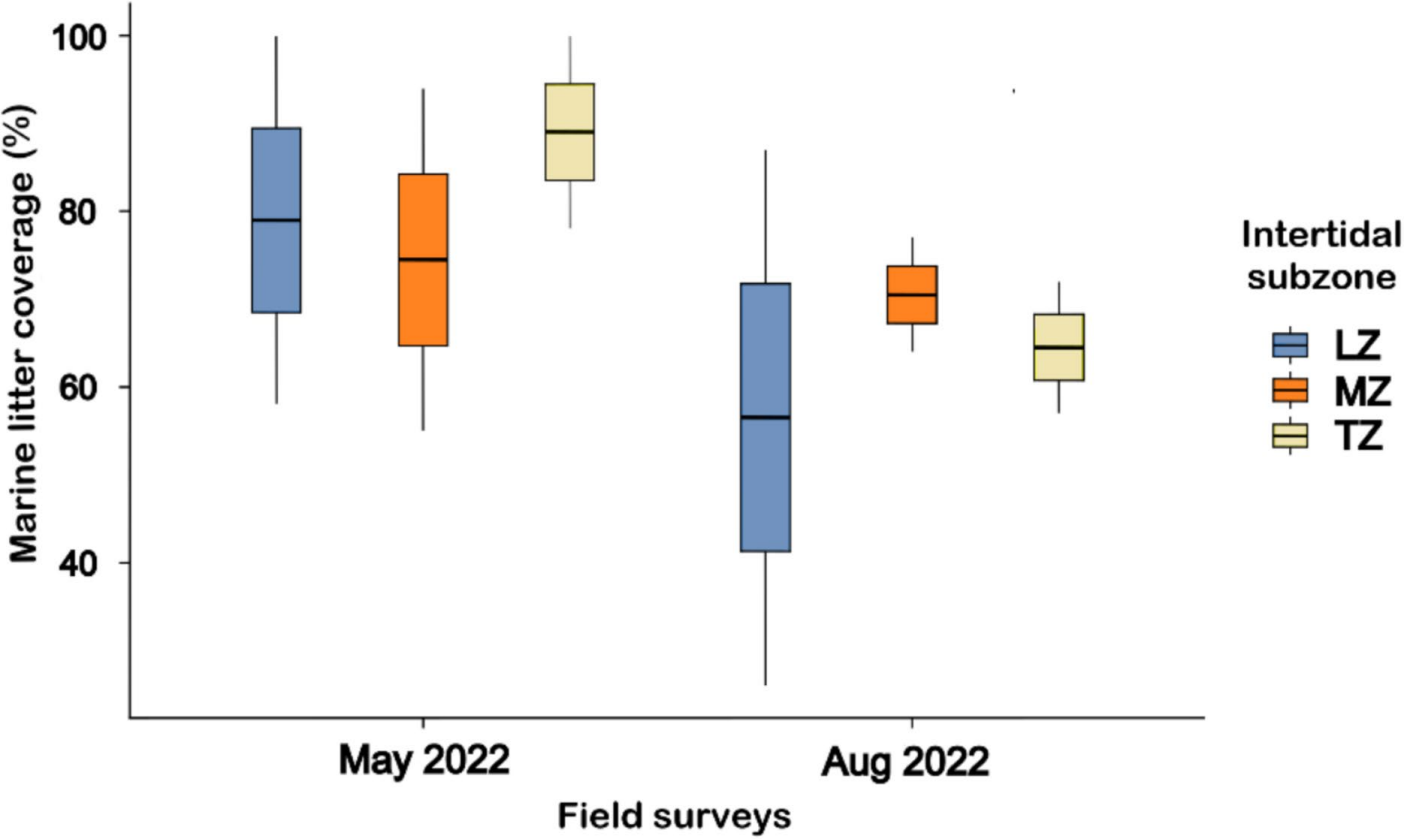
Boxplot with the marine litter coverage (percentage), in three intertidal subzones from Bom Jesus Cove mangrove swamp, Guanabara Bay, SE Brazil, where LZ (lower zone), MD (middle zone), and TZ (transition zone) represent subzones of the intertidal area

The marine litter coverage was 80.8 ± 7.4% in May 2022 (55.0 to 100.0%) and 63.8 ± 7.0% in August 2022 (26.0 to 87.0%) (Fig. 2). There was no significant difference between field surveys and subzones. The analysis revealed that between 71.1 and 94.6% of the marine litter was made of plastic or expanded polystyrene (Styrofoam™). The mean area covered was 7.3 ± 1.8 m^2^ and 5.7 ± 1.9 m^2^, respectively. The minimum value recorded was 2.34 m^2^ in the LZ in August 2022. The maximum was recorded as 9.0 m^2^ in one square in the TZ and one in the LZ, both observed in May 2022.

**Fig. 3:**
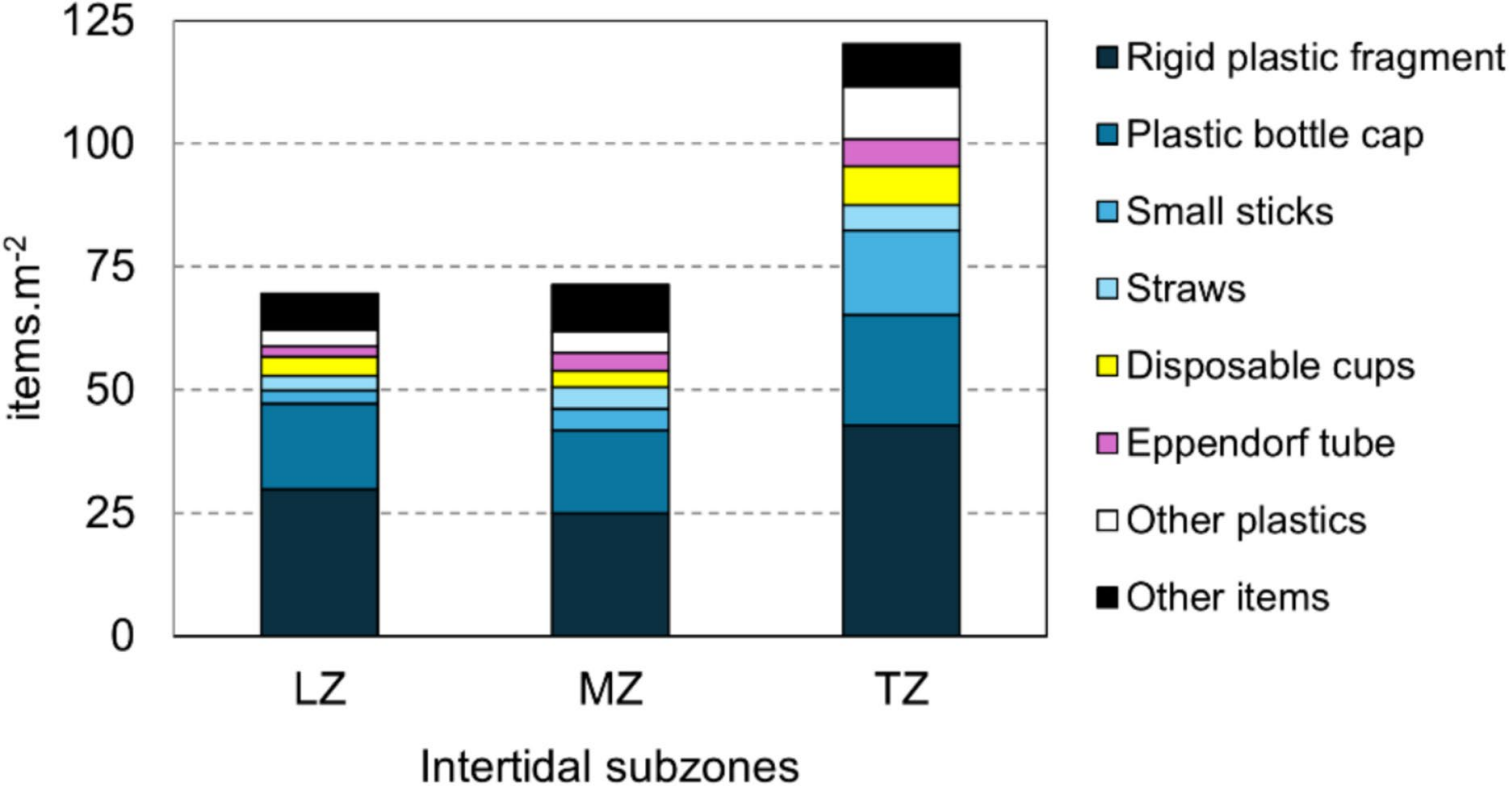
Types of macroscopic marine litter (items.m^−2^) in the surface substrate from Bom Jesus Cove mangrove swamp, Guanabara Bay, southeastern Brazil, where LZ (lower zone), MD (middle zone), and TZ (transition zone) represent subzones of the intertidal area

In the characterization of marine litter after collection (July 2022 only), the highest number of items.m^−^^2^ was observed in the transition zone (Table 1). Between 85.5% and 88.5% of the items collected were plastic or items predominantly composed of plastic, particularly rigid plastic fragments (32.6 ± 9.3 items.m^−2^), plastic bottle caps (18.8 ± 3.0 items.m^−2^), small sticks (8.1 ± 1.8 items.m^−2^), straws (4.1 ± 1.1 items.m^−2^), and disposable cups (5.0 ± 2.4 items.m^−2^) (Fig. 3). Other items included toys (dolls, balls, etc.), footwear and slippers, and Eppendorf tubes, which are in Rio de Janeiro associated with the sale and consumption of drugs, particularly cocaine.

**Table 1:** Quantity of macroscopic marine litter (in items.m^−2^) present on the substrate surface of the Bom Jesus Cove mangrove swamp, Guanabara Bay, Southeastern Brazil, where LZ (lower zone), MD (middle zone), and TZ (transition zone) represent subzones of the intertidal area. The acronyms employed in the “Chemical composition” column are defined as follows: MCC = multiple chemical composition, PP = polypropylene, PET = polyethylene terephthalate, PS = polystyrene, and HDPE = high-density polyethylene

Type	Chemical	Subzones		
	Composition	LZ	MZ	TZ
Rigid plastic fragment	MCC	29.8	25.0	42.9
Plastic bottle cap	PP	17.3	16.9	22.2
Plastic bottle	PET	1.7	2.9	2.3
	PP	0.0	0.0	0.2
Glass bottle	Glass	0.7	0.9	0.6
Small sticks (lollipop sticks, cotton swab, etc.)	PP	2.8	4.3	17.3
Straws	PP	2.9	4.2	5.1
Disposable cups	PP; PS	3.9	3.4	7.8
Plastic container lids	MCC	0.0	0.0	6.4
Cereal bags	PP	0.0	0.1	0.1
Pens	PP; HDPE	1.0	0.6	1.0
Toys (whole or parts)	MCC	2.0	3.3	3.7
Shoes and slippers	MCC	2.8	3.1	2.4
Related to drug trafficking (Eppendorf tube)	PP	2.2	3.6	5.6
Cigarette lighters	MCC	1.6	1.1	1.6
Syringe with needle	MCC	0.6	0.3	0.3
Unused condoms	Latex. other plastics	0.2	0.3	0.6
Aerosol deodorant bottle	Metal; PP	0.1	0.1	0.0
Textiles	Natural or synthetic	0.1	0.1	0.2
Other items	MCC	0.0	1.0	0.0
Styrofoam fragments^1^	PS	-	-	-
Total (except Styrofoam)	-	69.6	71.3	120.3

^1^During collection and sorting there was a lot of fragmentation, leading to inaccurate counting

### Identified species of crabs

Nine species of semi-terrestrial crab were recorded. The most frequently observed species were *Minuca rapax* (Smith, 1870), *Leptuca uruguayensis* (Nobili, 1901), and *U. cordatus*, which were also the most prevalent within the sample area. The remaining species were present in low quantities. Other fiddler crabs found were *Minuca victoriana* (von Hagen, 1987) (*n* = 2) and *Minuca burgersi* (Holthuis, 1967) (*n* = 1). Under fallen trees in the transition area between the intertidal and the supralittoral zones, we found two specimens of *Armases angustipes* (Dana, 1852). In wet parts of the intertidal zone, the species *Goniopsis cruentata* (Latreille, 1803) (*n* = 1) and *Neohelice granulata* (Dana, 1851) (*n* = 3) were recorded. In the slopes surrounding the mangrove swamp, in the supralittoral zone, we recorded the blue land crab *Cardisoma guanhumi* Latreille in Latreille, Le Peletier, Serville & Guérin, 1828 (about 16 active burrows and only one captured).

### Population assessment

#### *Ucides cordatus*

Most of the burrows observed were active, with a relative frequency ranging from 48.3% (May 2022) to 76.5% (August 2022). It was notable that a considerable proportion of the burrows were inactive, particularly in May (24.1%). The mean population density was 0.41 ± 0.69 ind.m^−2^ in May, and 0.61 ± 0.52 ind.m^−2^ in August. The crab size estimation revealed a CW variation between 35.8 and 115.6 mm (59.0 ± 16.9 mm). In May, the average was 62.6 ± 15.7, and in August, 57.7 ± 20.5 mm. Only one ovigerous female was observed in November 2021 (preliminary survey). No significant differences in population density and estimated CW of *U. cordatus* were observed between subzones or field surveys.

#### Fiddler crabs

**Fig. 4:**
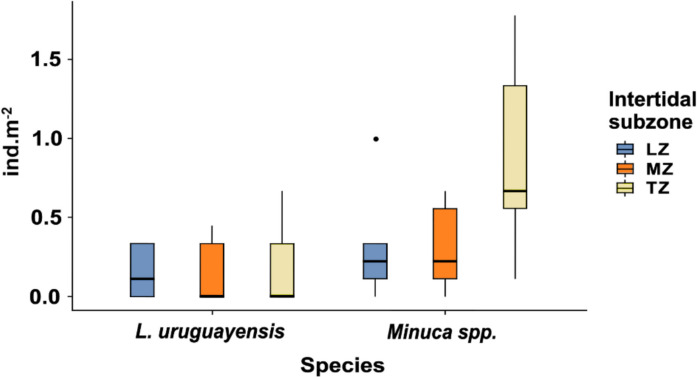
Boxplot of the population density (individuals per m^2^) of *Minuca* spp. and *L. uruguayensis* in three intertidal subzones from Bom Jesus Cove mangrove swamp, Guanabara Bay, SE Brazil, where LZ (lower zone), MD (middle zone), and TZ (transition zone) represent subzones of the intertidal area

We elected to maintain *Minuca* at the genus level due to the challenges associated with differentiating *M. rapax* from *M. victoriana* in situ. Among the fiddler crabs, this genus was the most abundant (Fig. 4). The mean abundance was 0.33 ± 0.39 ind.m^−2^ in the lower zone, 0.31 ± 0.29 ind.m^−2^ in the middle zone, and 0.89 ± 0.66 ind.m^−2^ in the intertidal transition zone. The values for *L. uruguayensis* were found to be similar in the lower and middle zone (0.16 ± 0.17 and 0.16 ± 0.22 ind.m^−2^, respectively), with the highest abundance observed in the transition zone (0.20 ± 0.30 ind.m^−2^). No significant differences in population density of fiddler crabs were observed between subzones. No ovigerous females were observed.

The data regarding the size of the specimens are presented in Table 2. The CW of *Minuca* spp. exhibited no significant variation between subzones, irrespective of sex. However, both sexes of *L. uruguayensis* exhibited significantly higher CW in the lower zone compared to the middle zone (Tukey, *p*: 0.01). The sex ratio (M:F) of *L. uruguayensis* was recorded as 1.0:1.0 in May and 1.0:0.5 in August.

**Table 2:** Mean ± standard deviation, minimum–maximum, and values of male (M) and female (F) carapace width (in mm), of *Minuca* spp. and *L. uruguayensis* from Bom Jesus Cove mangrove swamp, Guanabara Bay, SE Brazil

Taxa	Field	Sex	CW (mm)	
	Survey		Mean ± St.d	Min–Max
*Minuca* spp.	May	M	13.6 ± 2.8	6.0–20.9
		F	12.9 ± 1.7	8.3–15.8
	August	M	14.0 ± 3.8	6.0–21.0
		F	13.6 ± 3.3	7.0–19.0
*L. uruguayensis*	May	M	7.6 ± 1.0	5.8–9.4
		F	7.7 ± 1.5	5.3–10.3
	August	M	8.4 ± 1.3	6.0–10.0
		F	8.3 ± 1.6	6.0–10.0

### Diversity

In some quadrats, only one species was recorded (typically *U. cordatus*), thus rendering it impossible to calculate the diversity. In those squares where two or more species were recorded, the mean diversity was 1.05 ± 0.31 bits, with a range of 0.32 to 1.46 bits.

### Effects of macroscopic marine litter on crabs

**Fig. 5:**
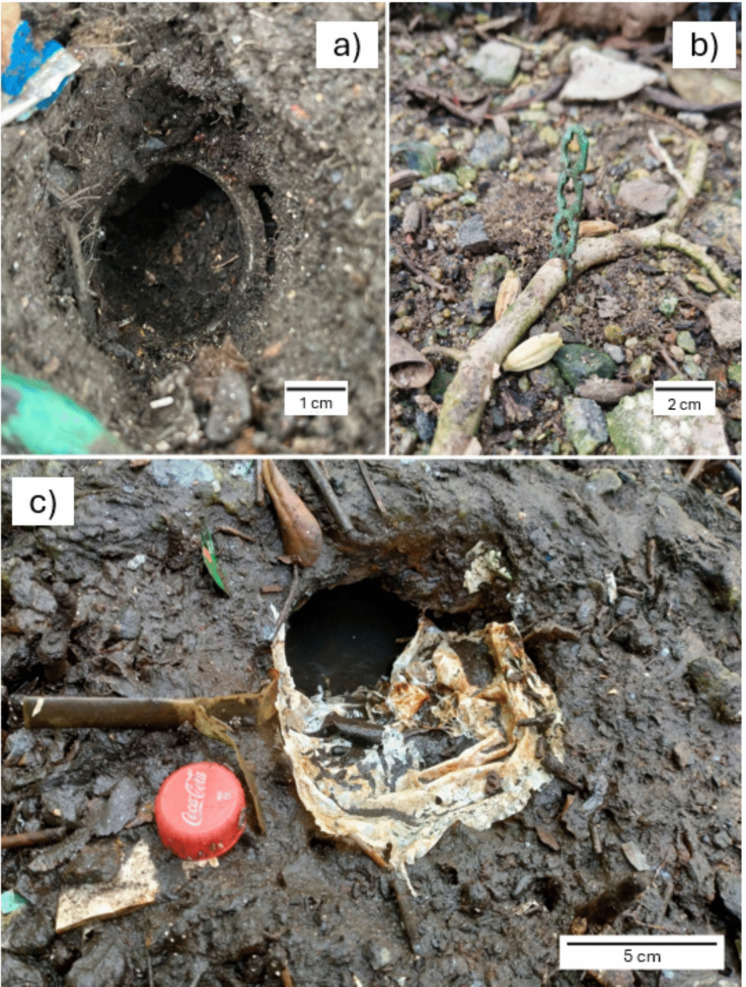
Macroscopic marine litter observed in the substrate of the Bom Jesus Cove mangrove swamp, Guanabara Bay, SE Brazil: **a -** part of the mouth of a plastic bottle (apparently PET) in the entrance of a fiddler crab burrow; **b -** a plastic popsicle stick entangled in *Talipariti pernambucense* root; **c -** a plastic bag in the entrance of a *Ucides cordatus* burrow. The images presented herewith were captured by the authors

In the study area, despite the frequent movement of the residues by tides, a considerable number of macroscopic marine litter were observed to be fixed in the substrate, within burrows, or attached to roots (Fig. 5). These residues remained in the same position for an extended period during the research. A limited number of entanglements of crabs were recorded. We observed one *U. cordatus* entangled in plastic filaments (from a cereal bag) and another in a tangle of fishing line attached to the roots. We also observed one died *U. cordatus*, due to obstruction of this burrow mouth by a rigid plastic box (in Brazil named “engradado”, used as beer transport). For reasons that remain unclear, the crab was unable to extricate itself from the situation.

**Fig. 6:**
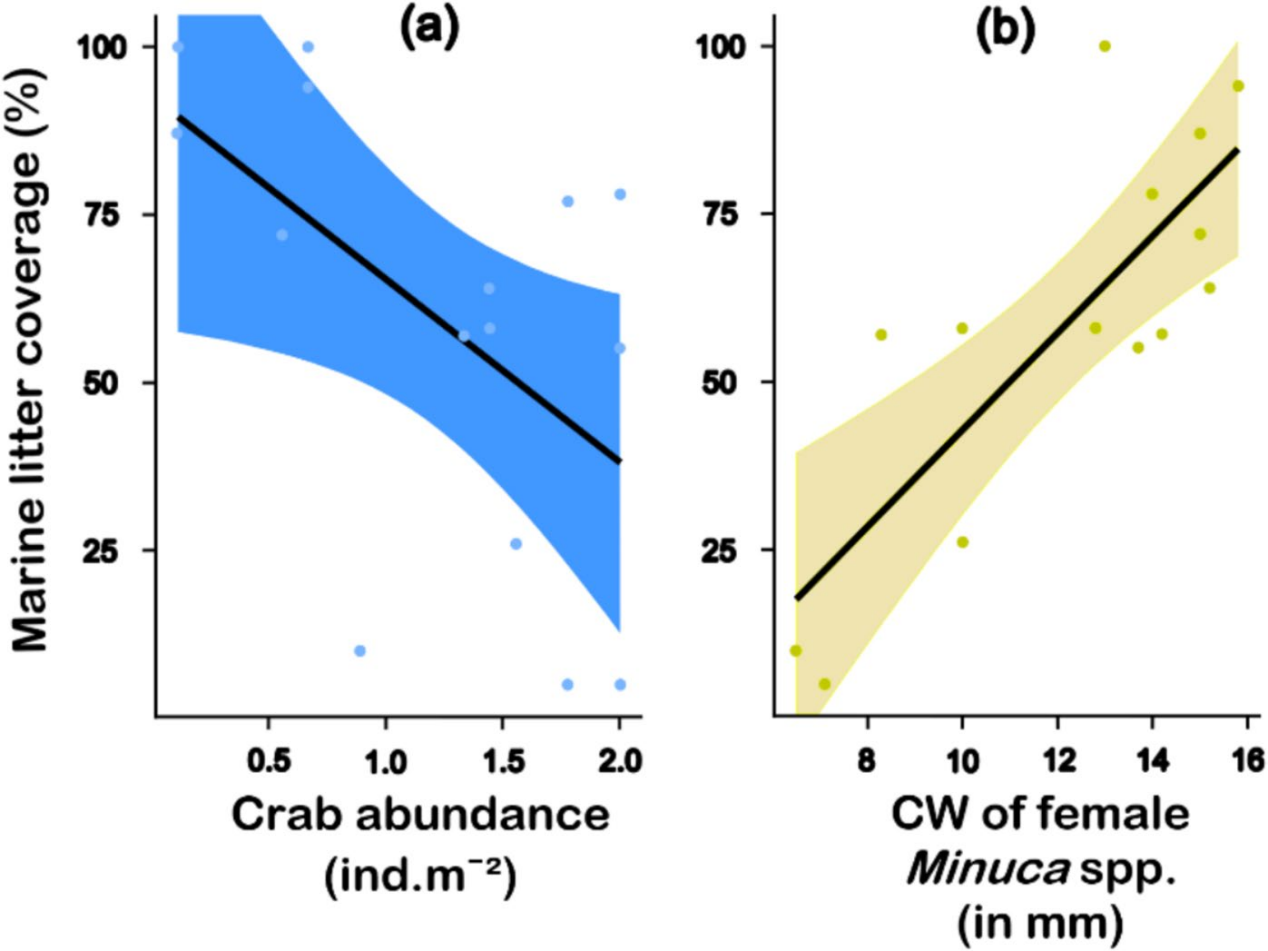
**a -** Correlation between the marine litter coverage (percentage) and the total abundance of crabs (in ind.m^−2^) (Pearson, *R* = −0.63; *p* = 0.03) and **b -** Correlation between the marine litter coverage and the carapace width (CW, in mm) of female *Minuca* spp. (Pearson, *R* = 0.80; *p* < 0.001). The crabs were collected in Bom Jesus Cove mangrove swamp, Guanabara Bay, SE Brazil. See the subsection “Impacts of marine litter on crabs” in the Discussion section for more information on the hypotheses regarding these correlations

No significant correlation was observed between diversity and marine litter coverage (Pearson, *R* = −0.096; *p* = 0.73). However, a negative correlation, moderate and statistically significant, was observed between marine litter coverage and the total population density of crabs (Pearson, *R* = −0.63; *p* = 0.03) (Fig. 6a). Considering crab’s size, there was a notable positive correlation between marine litter coverage and the estimated CW of *U. cordatus* (Pearson, *R* = 0.83; *p* = 0.02). The same pattern was seen with the CW of *Minuca* spp. females (Pearson, *R* = 0.80; *p* < 0.001) (Fig. 6b).

## Discussion

### Marine litter

Mangroves, situated in the coastal zone and subject to the influence of tidal flows and river discharges, are one of the ecosystems most impacted by marine litter. This is due to the presence of trees with aerial roots and the prevalence of crab burrows, which retain both macroscopic and microscopic marine litter (Riascos et al., 2019). In the present study, a high quantity of macroscopic marine litter was observed on the west side of one of the most anthropogenically altered bays in the world. The abundance observed in this study, ranging from 69.9 to 120.3 items.m^−2^ was similar to but higher than those reported by Duarte et al. (2023) in Santos, Southeast Brazil. In Santos, the authors recorded 52.09 items.m^−2^ in a mangrove swamp near a major commercial port, surrounded by a considerable number of industrial facilities and an extensive irregular urban occupation. The values observed in the present study also were considerably higher than reported in other mangroves from Brazil (e.g., Cordeiro & Costa, 2010), from the Colombian Pacific coast (Riascos et al., 2019), from East Africa (Okuku et al., 2023), and in South China (Sun et al., 2023; Vorsatz et al., 2023). The elevated abundance observed in this study is indicative of both the oceanographic characteristics of the bay and the inadequate waste management employed in the city of Rio de Janeiro and other municipalities surrounding Guanabara Bay. As expected, in our study we observed a predominance of plastics (85.5 to 88.5%), with an emphasis on rigid fragments. Duarte et al. (2023), studying a mangrove swamp in SE Brazil, found that 62.3% of the marine litter was plastic, followed by Styrofoam (30.5%). These authors identified the prevalence of marine litter of domestic origin, except for plastic microtubes (presumably Eppendorf tubes) and syringes. As demonstrated in the relevant literature, plastics were the predominant marine litter observed in mangroves in western Colombia (Riascos et al., 2019), East Africa (Okuku et al., 2023), and southern China (Vorsatz et al., 2023).

The abundance of plastics in mangroves is related both to their low density, which facilitates transport by water and wind, and to the way waste is managed in each location. It is also related to the type of use, with single-use plastics being a major problem (Pearson et al., 2022). Indeed, some of the most common items found at the MBJC were plastic bottle tops, small sticks, straws, and disposable cups, all of which were domestic and single-use items. Most of the rigid fragments observed might have resulted from the degradation of larger items such as cups, bottles, appliances, electronics, and toys. It is established that macroscopic plastics have the potential to transform into microplastics (Manzoor et al., 2022), which can inadvertently be ingested by crabs, as has been comprehensively evidenced (see Pisani et al., 2022). However, while plastic is macroscopic, the impacts are different. Macroscopic plastics have been observed to cause changes in the environment, including the obstruction of substrates and burrows, and the entanglement of crabs.

In our study area we observed high marine litter coverage, with a maximum of 100.0% in May 2022 and 87.0% in August 2022. We observed that the area covered varied between 4.95 and 9.0 m^2^ in May and between 2.34 and 7.83 m^2^ in August. For comparison, the highest percentages of substrate coverage observed by Bulow and Ferdinand (2013) were close to 50%, in mangroves on the Pacific coast of Panama. Vorsatz et al. (2023), looking at the average area covered by debris (in m^2^), found a value of 0.11 m^2^ in mangroves in the Hong Kong (Southern China). Considering the marine litter coverage and abundance of litter, the MBJC appears to be one of the environments most affected by marine litter in Brazil and worldwide. As we pointed out, the differences can be explained by a combination of public policies, floristic composition, and oceanographic issues.

### Population parameters and diversity

#### *Ucides cordatus*

The CW estimate for *U. cordatus* was similar to that reported in the literature for mangroves from north to south Brazil, the country with the largest distribution of this species. The average CW observed in the present study (59.0 ± 16.9 mm) was slightly lower than that observed in the Brazilian North (Amaral et al., 2014) and in the Southeast (Pinheiro, 2006), in areas dominated by *Avicennia*. Based on the compilation presented by Mota et al. (2023), with about 17 populations/subpopulations in Brazil, our value was higher than that observed in some mangroves in the northeast (*n* = 4) and southeast/south (*n* = 6). According to these authors, there is no evidence of a correlation between the average size of *U. cordatus* and latitude. Previous studies have shown the variation in CW is mainly related to the degree of local flooding (due to the area and time available for foraging), the dominant mangrove tree species, the organic matter content of the sediment, the presence or absence of fishing, among other factors. The species’ preference for mixed litter, especially *Rhizophora* and *Avicennia* leaves (predominant in our study area) (Christofoletti et al., 2013), for sediments with high organic matter content (Lima et al., 2023), and the absence of fishing in the MBJC may be related to the relatively large average size estimated in the present study.

Regarding the population density of *U. cordatus*, our average (0.5 ± 0.6 ind.m^−^^2^) was lower than that observed in most Brazilian mangroves (Schmidt et al., 2008; Goés et al., 2010; Sandrini-Neto & Lana; 2011; Oliveira et al., 2013; Amaral et al., 2014; Aviz et al., 2020; Lima et al., 2023; Mota et al., 2023). However, it was similar to that observed in the mangroves of the Eastern Guanabara Bay Conservation Units (0.41 ± 0.19 ind.m^−2^— Costa et al., 2014). Some environmental factors that determine the average size of crabs also influence their abundance. Certainly, variations in the quality and quantity of the litter, the extent of the intertidal zone and the degree of daily oscillation of the sea level, the characteristics of the sediment, and the presence or absence of fishing in a locality are all relevant factors. At the MBJC, the *U. cordatus* population suffers from additional anthropogenic pressures, particularly pollution. Although there is no significant fishing at the site, high levels of macroscopic litter (presented in this study), high concentrations of metals in the channel adjacent to the study area (Borges et al., 2014), high contamination by other pollutants (reviewed by Soares-Gomes et al., 2016), including microplastics (Olivatto et al., 2019), are relevant aspects that may be related to the low abundance of *U. cordatus*.

### Fiddler crabs

The carapace width of *Minuca* spp., males and females, was similar to that observed in other mangroves in southeastern Brazil, both preserved and impacted (reviewed by Ribeiro & Bezerra, 2014). The same was observed in *L. uruguayensis*, females and males, which showed similar values to other populations (Ribeiro & Bezerra, op cit).

Discussing fiddler crab abundance can be challenging as many studies only report absolute numbers of individuals without adequately defining the capture area (e.g. in m^2^). Methodological differences are also a limitation, with some studies reporting population densities based on counting burrows and others based on capturing individuals (Johnson, 2003; Colpo & Negreiros–Fransozo, 2016). Considering only studies with similar methodologies, the mean density of *Minuca* spp. in our study was 4 to 6 times lower than that observed with *M. rapax* in other mangroves in southeastern Brazil. Carvalho et al. (2018) observed an average of 3.40 ind.m^−2^ and Checon and Costa (2017) of 2.28 ind.m^−2^. For *L. uruguayensis*, we observed the same trend, with even more discrepant values. We found an average of 0.17 ± 0.22 ind.m^−2^, but other authors have observed values of 3.95 ind.m^−2^ (Checon & Costa, 2017), 11.87 ind.m^−2^ (Arakaki et al., 2020), 32.65 ind.m^−2^ (Borges et al., 2024) and 38.80 ind.m^−2^ (Machado et al., 2013). Checon and Costa (2017) and Stauffer (2024) observed higher densities of *L. uruguayensis* in the intertidal lower zone, which is more inundated. In our study, there was no notable variation in the abundance of *L. uruguayensis*, despite the differences in water content and organic matter concentrations observed between the intertidal zones. Like *U. cordatus*, the lower abundance of fiddler crabs in the MBJC may be related to the high levels of macroscopic litter and other contaminants.

### Diversity

The richness and diversity of semi-terrestrial crabs in the MBJC were low. Although there are no recent surveys of mangrove crab species in Rio de Janeiro State, Schmidt and Diele (2023) suggest that up to 19 species of semi-terrestrial crabs occur in the mangroves of this part of the Brazilian coast. Of the nine species recorded, only *U. cordatus*, *L. uruguayensis*, and *Minuca* spp. exhibited high abundance. Castiglioni & Negreiros-Fransozo (2006) highlighted the abundance of *M. rapax* in anthropized or vegetation-free mangroves. According to Bedê et al. (2008), *M. rapax* and *Leptuca leptodactyla* were very versatile species, occurring in a wide variety of sediments with different concentrations of organic matter, with or without vegetation.

It is worth noting in the present study that we observed an absence of some species that are commonly found in mangroves in southeastern Brazil. The most notable case is the absence of *Aratus pisonii*, an arboreal and herbivorous crab. It is possible that there are floristic, sedimentological, hydrological or oceanographic features that prevent the species from settling and recruiting. Further research is needed. Interestingly, another notable absence is the lack of Diptera species typically found in other mangrove areas of the bay, such as “maruins” (Ceratopogonidae) and “mutucas” (Tabanidae). As the larvae of Ceratopogonidae mosquitoes depend on wet environments for their development (Ferreira Keppler et al., 2014), their absence is another indicator of the restrictive conditions of the substrates and water bodies of the MBJC.

### Impacts of marine litter on crabs

The percentage of inactive burrows in MBJC was high, reaching 24.1% in May 2022. In a two-year monitoring study of *U. cordatus*, Gonçalves et al. (2022) frequently found percentages of inactive burrows below 10%. However, after the occurrence of a cyclone, rates of more than 20% were observed. The substantial number of inactive burrows identified in the present study may serve as an indicator of a deterioration in environmental quality, similar to that observed by Gonçalves et al. (2022).

In the present study many marine litter were found attached to the mangrove roots, integrated into the sediment, or forming part of the entrance to the burrows (as seen in Fig. 5). As previously stated, the elevated levels of pollution in the study area, in conjunction with the considerable presence of macroscopic marine litter, may be contributing factors to the abandonment of burrows or the mortality of crabs. Iribarne et al. (2000) and Luo et al. (2021) raise the issue of marine litter obstructing crab burrows. The burrow shape can facilitate the retention of marine litter, as seen in a study of the crab *Neohelice granulata* (Iribarne et al., 2000). To date, no investigation into the interior of the burrows has been conducted in MBJC. However, it is probable that marine litter is present on the inner portion, and that this is a contributing factor to the deterioration of burrow conditions.

We noticed significantly fewer crabs in areas with more marine litter coverage. The same was seen by Bulow and Ferdinand (2013), with a reduction in the number of fiddler crab burrows in areas with greater marine litter coverage (*R*^2^ = 0.33, *p* < 0.008). The reduction in the number of crabs caused by marine litter is of great concern, especially given the ecological role of these animals. According to Nordhaus et al. (2006), *U. cordatus* can consume up to 81.3% of mangrove leaf production. The authors suggest that the species’ intensive processing of litter helps to retain nutrients and energy within the ecosystem. As reviewed by Kristensen (2008), the activity of intertidal crabs is also of great importance, as their feeding and burrowing activities have several significant effects on the environment, such as creating ecological niches for microbiota; redistributing and burying organic matter; increasing heterotrophic microbial activity in the sediment; nutrient conservation; increased sediment–water-air exchange; passive irrigation; altered near-surface redox conditions; increased availability of oxidized iron; reduced microbial biomass; reduced near-surface heterotrophic microbial activity; and enhanced subsurface microbial iron reduction. Therefore, the reduction of key species such as *U. cordatus* and fiddler crabs caused by litter accumulation in mangroves can be highly detrimental to the entire ecosystem.

In addition to the decline in the number of crabs, for the first time a positive correlation was observed between the marine litter coverage and the CW of *U. cordatus* and *Minuca* spp. females. The study area is subject to a variety of pressures (see the “Study area” section), not only due to the significant presence of marine litter. In order to avoid conclusions based on mere coincidence, further studies will be necessary. However, the absence (or near absence) of fiddler crabs in areas with higher marine litter coverage was a notable observation. It is important to note that some probable causes are unrelated to the issue of marine litter. For *U. cordatus*, the absence of fishing in the designated study area has been previously documented. This observation may provide a rationale for the comparatively elevated mean CW recorded in the MBJC. An alternative hypothesis, which encompasses both species, relates to sediment properties. As Riascos and Gomez (2023) observed, the size of the *Minuca vocator* females (ovigerous and non-ovigerous) increased slightly in an urban mangrove. The authors hypothesize that despite the numerous anthropogenic disturbances in urban mangroves, the enrichment of sediment with organic matter may be conducive to the size of *M. vocator*. In the present study, the variation in organic matter and water in the sediments of MBJC was not reflected in the size of *U. cordatus* and *Minuca* spp. females. As demonstrated, there was no significant variation in CW of these species in the subzones.

In the event that further evidence is discovered that marine debris coverage exerts a selective effect on crab size, a list of probable reasons can be provided. It has been demonstrated that marine debris not only covers a substantial area of the substrate, thereby reducing the available foraging area, but also enters burrows and can prevent them from functioning. This phenomenon has been observed by Bulow and Ferdinand (2013), Iribarne et al. (2000), and Luo et al. (2021). It has been demonstrated that marine debris can adhere to roots and sediments, thus remaining in the mangrove for extended periods, as outlined in the present study and by Martin et al. (2019). It can be hypothesized that the marine litter coverage may impose a greater physical challenge for mangrove crabs in terms of mobility, food acquisition, burrow excavation and maintenance. The additional energy expenditure required on a daily basis appears to contradict the selection of larger crabs. Nevertheless, in areas where population densities are lower and marine debris is more abundant, competition is reduced, thus allowing larger individuals with greater strength the opportunity to deal with the marine litter. It is important to note that this hypothesis is still in its preliminary stages and that further studies, including experimental ones, will be needed to prove or disprove it. It is not possible to disregard the possibility that macroscopic marine litter has more deleterious effects on smaller species or individuals.

## Conclusions

The present study analyzed populations of semi-terrestrial crabs in one of the most polluted areas in southeastern Brazil. Contamination by macroscopic marine litter of anthropogenic origin was detrimental to the crabs, resulting in a decline in population numbers, entrapment, occlusion of feeding areas, and obstruction of burrows. Furthermore, a propensity for elevated CW values in *U. cordatus* and female *Minuca* was documented in regions characterized by increased marine litter coverage. It is imperative that further research be conducted on this trend, with a particular focus on other regions that are similarly afflicted by macroscopic marine litter. Should this hypothesis be corroborated by subsequent research, it would be a matter of significant concern. The observed tendency to favor larger species or larger individuals may be related to the decline in diversity of semi-terrestrial crabs, particularly in mangroves, which are most affected by marine litter.

In relation to the efforts of the *Orla sem Lixo* Project, monitoring of crabs will continue. A new series of experiments is being planned that will examine the interaction of crabs with marine litter. On March 11, 2026, barriers designed to impede the movement of floating marine litter were erected around the entire perimeter of the MBJC. It has been determined that cleanup actions shall be initiated in the forthcoming years. A primary concern in this process is the mitigation of any potential adverse impacts on the local crab population.

## Data Availability

The data for this work can be requested via email to the corresponding authors.
